# Does effectiveness in performance appraisal improve with rater training?

**DOI:** 10.1371/journal.pone.0222694

**Published:** 2019-09-19

**Authors:** Christian Rosales Sánchez, Dolores Díaz-Cabrera, Estefanía Hernández-Fernaud

**Affiliations:** Universidad de La Laguna, Tenerife, Islas Canarias, España; Sapienza, University of Rome, ITALY

## Abstract

Performance appraisal is a complex process by which an organization can determine the extent to which employees are performing their work effectively. However, this appraisal may not be accurate if there is no reduction in the impact of problems caused by possibly subjective rater judgements. The main objective of this work is to check the effectiveness—separately and jointly—of the following four training programmes in the extant literature aimed at improving the accuracy of performance assessment: 1) Performance Dimension Training, 2) Frame-of-Reference, 3) Rater Error Training, and 4) Behavioural Observation Training. Based on these training strategies, three programmes were designed and applied separately. A fourth programme was a combination of the other three. We analyzed two studies using different samples (85 students and 42 employees) for the existence of differences in the levels of knowledge of performance and its dimensions, rater errors, observational accuracy, and accuracy of task and citizenship performance appraisal, according to the type of training raters receive. First, the main results show that training based on performance dimensions and the creation of a common framework, in addition to the training that includes the four programmes (Training_4_programmes), increases the level of knowledge of performance and its dimensions. Second, groups that receive training in rater error score higher in knowledge of biases than the other groups, whether or not they have received training. Third, participants’ observational accuracy improves with each new moment measure (post-training and follow-up), though not because of the type of training received. Fourth, participants who receive training through the programme that combine the other four gave a task performance appraisal that was closer to the one undertaken by the judges-experts than the other groups. And finally, students’ citizenship performance appraisal does not vary according to type of training or to different moment measures, whereas the group of employees who received all four types of training gave a more accurate citizenship performance assessment.

## Introduction

Task and citizenship performance appraisal is essential for organizations because it provides important information for people management [1]. Task performance appraisal is centred on the contribution of specific employees in terms of tasks formally assigned to their job and position [2,3,4]. Citizenship performance appraisal refers to behaviours that provide assistance and support for the basic formal tasks of the job and the creation of an organizational environment of social and psychological support, which fosters the smooth running of the organization [5,6]. This paper focuses on task and citizenship performance because these performances are generally evaluated by all organizations that follow a performance appraisal system, unlike counterproductive behaviours or the most recently proposed adaptive performance. Checking the effectiveness of different training programmes involves considering both task and citizenship performance, because they are the most commonly used in practice and because very few studies have analyzed the effectiveness of training on citizenship performance appraisals.

An efficient performance appraisal process calls for rater accuracy. The main objective of this work is therefore to check whether the theoretical and practical training of performance raters improves the accuracy of their appraisal. Traditionally, two strategies have been used in response to the problems created by subjective rater judgements on performance: more accurate rating scales and rater training [7]. In recent years, interest has centred on rater training, since improved rater response scales are not considered to further increase performance accuracy or reduce bias [8]. Training encourages raters to use the skills and tools required to improve their performance accuracy, while increasing participant satisfaction with the system [9].

Woehr and Huffcutt [7] categorized existing rater training programmes and carried out a subsequent meta-analysis, based on the results of previous studies, to assess their effectiveness, classifying them into four types: 1) Rater Error Training (RET); 2) Performance Dimension Training (PDimT); 3) Behavioural Observation Training (BOT); and 4) Frame-of-Reference (FOR). These four strategies share the same objective: to improve rater precision and accuracy.

Verifying the effectiveness of training programmes to improve rating accuracy entails several procedures to compare rater rating with true scores (or true scores estimate, a term proposed by Sulsky & Blazer [10] as being more precise), generally issued by experts (for a review, see Sulsky & Blazer [10]). The most commonly used measurements by which to operationalize true scores are the four indices proposed by Cronbach [11] and the two by Borman [12]. These indices entail comparing the distance between the scores given by a group of experts (true scores estimates) and those given by training programme participants [13].

Studies on rater training programmes have generally used one of Cronbach’s [11] or Borman’s [12] indices, or a combination of several. Thus, Gorman and Rentsch [14,15], Sulsky and Day [16], Sulsky and Kline [17], Raczynski, Cohen, Engelhard, and Lu [18], and Woehr [19], among others, use Cronbach’s indices [11], along with Borman’s distance accuracy [12], to analyze training effectiveness.

With regard to the four types of training classified by Woehr and Huffcutt [7], the main objective of training in RET is to increase appraisal accuracy by familiarizing raters with common classification errors and biases (e.g. similarity, contrast, primacy, recency, negativity, first impression, leniency, central tendency, severity, halo effect) [9,20,7]. Traditionally, participants are trained in the definitions of the involuntary biases in which they may incur and which affect the accuracy of their appraisals. To that end, they are shown graphic illustrations of numerical examples of how such biases may interfere with their appraisals. Moreover, some programmes include debates with participants about how to avoid bias in performance appraisals of fictitious characters shown in videos [21]. The results of several studies indicate that this programme reduces the influence of these biases on appraisal [20,21,22]. However, results also show that this reduction may have a negative effect on rater accuracy [23], depending on the location of the main focus of the training [7].

Smith’s [24] PDimT emerges as an alternative to accuracy problems in bias training. The main aim is to improve rater accuracy by familiarizing raters with the meaning of performance, along with its components and dimensions, and by involving them in the design and review of the rating scale being used [24,7]. Results show that this approach increases the degree of agreement between the appraisals of several raters and between those made by each of them [10], thereby ensuring assessments are more precise and accurate [19]. Nevertheless, we found no study that assesses the influence on rater accuracy of this programme alone. Thus, the study by Pulakos [25], which compared the RET and PDimT programmes, reveals, among other results, that the group that only received training in performance dimensions gave more precise scores than the untrained group.

Third, in 1980, there emerged a new line of research in training programmes focusing on rater observation skills (BOT) [26]. The objective was for raters to closely observe ratee behaviours and to improve their own recall of them [9,27]. This strategy uses memory and recognition of specific behaviour events as a dependent variable [28,26]. Thus, several techniques and procedures have been evaluated [9,16,26]. Aguinis [9] proposes training raters to use notes or diaries as observation strategies to enable them to record behaviours that must be evaluated in each performance dimension. Sulsky and Day [16] include measures of behavioural recognition, in which participants are asked to indicate from a list which behaviours really occurred in a fictitious situation. And Thornton and Zorich [26] created a multiple-choice questionnaire, with true/false responses, or a combination of alternatives, to evaluate a sample of behaviours with stimulus material. Although the effectiveness of this type of training has been little studied, the meta-analysis made by Woehr and Huffcutt [7] shows a positive effect on both appraisal and observational accuracy (*d* = .77 and .49, respectively). However, the study by Hedge and Kavanagh [29] does not provide conclusive results on the effectiveness of this programme.

Fourth, the frame-of-reference strategy (FOR), proposed by Bernardin and Buckley [30], highlights the importance of the fact that raters a) are aware of the multidimensionality of performance, in order to familiarize themselves with identifying each ratee behaviour with the correct performance dimension [28,16], and that they b) share a framework or common conceptualization regarding the nature of performance, so that it can be evaluated in a similar way by different raters [31,13]. It therefore focuses on intervening in the way in which raters codify, organize, and recall information [32]. The final aim is to obtain more accurate appraisals from participants based on the presentation of small samples of performance at work, along with the performance dimension appraisal issued by a group of experts [24]. However, variations have been observed in the studies, in terms of experimental design, programme structure, length of training, and method of accuracy appraisal. Thus, the programme designed by Bernardin and Buckley [30] establishes a series of stages, ranging from familiarizing participants with how to obtain a profile of personal requirements for jobs, based on job descriptions, a performance appraisal of a fictitious employee, and a justification of the appraisal, to a group debate about the discrepancies between the correct appraisals provided by the trainer and those issued by the participants.

In recent years, the FOR programme has been the most used and cited by various authors compared with other strategies [33,34,14,15,35,36,37,18,13,38,28,16], although variations have been observed in the studies regarding experimental design, programme structure, length of training, and method of accuracy appraisal. Studies have shown the positive effect of the FOR programme on appraisal accuracy [39,14,40,27,13,28,16]. Thus, Lievens and Sánchez [36] found that the trained group presented significantly higher values in disciminant validity, interrater reliability, and appraisal accuracy compared with the control group.

However, there were variations in the results regarding appraisal accuracy (*d* = .50 and *d* = .83, respectively) in the studies by Roch et al. [13] and by Woehr and Huffcutt [7]. As Roch et al. [13] suggest, this variation may be due to sample size, number of effect sizes, and the measurements used to determine the degree of accuracy. Finally, some research has examined a little studied variant in the field of rater training: programme combinations. On the one hand, it highlights comparison of the effect of the combined training of two rather than only one of the strategies [27,32], and on the other, the combination of all four main training types, but without comparing the effect of each training type independently [41].

The aim of this research is therefore to analyze how training influences rater performance appraisal according to the type of training received. We propose the following specific objectives:

1. To analyze how the level of knowledge of job performance, its dimensions, assessment, and the most common biases vary according to the type of training received.
2. To verify whether rater observational accuracy changes according to training type.
3. To examine whether the task and citizenship performance appraisal of a fictitious employee is modified according to the training programme followed.

To test the objectives, we carried out two studies.

## First study

In the first study we have proposed four hypotheses:

*Hypothesis 1*.*1* (*H1*.*1*): groups trained in performance dimensions and FOR, and in all four programme types will score higher in general knowledge of performance than others programmes. This hypothesis is based on the fact that the programmes PDimT and FOR concentrate on developing knowledge about performance and its dimensions [31,13,24,28,16,7]. Moreover, the programme that includes the contents of all four programmes focuses on participant familiarization with the concept of performance and its dimensions, because it incorporates the contents of PDimT and FOR.

*Hypothesis 1*.*2* (*H1*.*2*): groups trained in Rater Error Training (RET) and in all four programme types will score higher in knowledge of appraisal biases than the other groups. The RET training programme aims to develop participant awareness of the biases that can affect appraisal accuracy [7]. It is therefore logical to expect participants in this programme to have better mastery of biases and how to avoid them than the other training programmes. This is also true for the complete training programme, because it includes training in bias identification.

*Hypothesis 1*.*3* (*H1*.*3*): group trained in observational accuracy and in all four programme types will identify more accurately the occurrence or non-occurrence of various events. The aim of the BOT training programme is to improve the observational capacity of participants regarding the behaviours of employees being appraised [9,27] and it is therefore expected to foster greater observational accuracy. The same is expected of the programme that combines all four types of training, since it includes training in rater observation skills from the BOT programme.

*Hypothesis 1*.*4* (*H1*.*4*): group trained in all four training programmes will produce a task and citizenship performance appraisal closer to the expert judgement than the other groups. Given that the different training programmes have obtained some results that endorse their effectiveness in enhancing appraisal accuracy in the specific aspects on which they focus [20,21,22,36,25,19,7], the inclusion of such content (observation, rater error, dimensions and frame-of-reference) in a single training programme should make this programme more effective.

## Method

### Participants

G*Power: Statistical Power Analyses revealed that sample size should be 75 people, with a 95% confidence level and 5% margin of error for five groups and five dependent variables. The sample was composed of 85 second-year psychology undergraduates, of whom 80.5% were women and 19.5% men. The average age was 20.5 years (*SD* = 3.21, range, 19–38 years). No student had prior work experience.

### Design

The design was quasi-experimental, factorial-multivariable and longitudinal (repeated measures), with three moment measures of the training received: before, on completion, and after a month (follow-up); the within-group variable was Moment measure.

As an independent between-group variable, Type of training had five groups: 1) training in Knowledge of dimensions and Frame-of-reference (KdFOR) (*n* = 18) combines the strategies of two programmes, PDimT and FOR, since both aim to improve knowledge of performance and its dimensions, by fostering correct assessment and agreement between raters, 2) Observational accuracy training (*n* = 16), 3) Rater Error Training (RET) (*n* = 19), 4) Training_4_Programmes, training that includes the content addressed in the other three groups (*n* = 15), and 5) Control group (no training, *n* = 17). The S1 Appendix contains the objectives, contents, and length of each training programme. The methodological strategies used in all four training programmes included group discussion, written practice, and the joint compiling of conclusions. Video instruction was also given in training programmes on observational accuracy, identification, risk prevention, and full training. Five measures were used as dependent variables: 1) Knowledge of performance and its dimensions, 2) Knowledge of biases in appraisals, 3) Observational accuracy, 4) Task performance appraisal, and 5) Citizenship performance appraisal.

### Materials and tools

The materials used included two video stimuli, five training videos, a short film, and training manuals. Five measuring tools were used: three ad-hoc questionnaires: two to evaluate knowledge of performance and its dimensions, and of bias in appraisal, and one to measure participants’ observational accuracy; and two performance assessment scales (task or citizenship). Below is a description of the materials and tools:

- **Videos**: two versions of one video were created as stimulus material for performance assessments. The video shows five samples of an employee’s performance over five working days. The work samples are the same in both videos, the script for which was created in a previous study [42]. These work samples included a series of performance task activities habitually carried out by administrative and office staff, as well as the employee’s citizenship behaviours. On three of the five working days, the employee gave an adequate task and citizenship performance, while on the two remaining days, performance in both areas was inadequate. To reduce the recall effect when the material was assessed three times, the working days and main actors were presented in a different order. Both versions of the videos lasted around 30 minutes.
- **Training videos**: in several training activities, five videos illustrated various performance samples of one or several employees in an administrative position.
- **Short film**: an 18-minute-long audio-visual film entitled *Life Vest Under Your Seat* was used to assess observational accuracy [43]. The short film is set on a flight from Madrid to Miami, whose flight path is altered because of the conduct of a passenger who decides to break all the usual in-flight rules of behaviour and safety. This short film was chosen because it had not been widely circulated and was largely unknown to participants, thereby counteracting the influence of recall.
- **Training manuals**: trainer and participant manuals were devised for each training programme. They included various individual, group, theoretical, and practical activities and exercises (S1 Appendix).
- **Questionnaire on knowledge of performance and its dimensions**: this ad-hoc paper tool was composed of 11 items that evaluated knowledge of job performance, types, and dimensions. Participants had to decide whether each item was true or false, obtaining a score from 0 to 10. This questionnaire is included as supplementary material (S1 Questionnaire).
- **Questionnaire on knowledge of biases in performance assessment**: this ad-hoc tool was composed of 21 items on the most frequently occurring biases in performance assessment. The response scale was dichotomous (True or False), with a score from 0 to 10. This questionnaire is included as supplementary material (S2 Questionnaire).
- **Checklist of observational accuracy**: a 155-item ad-hoc paper tool that describes events that may or may not have happened in the short film *Life Vest Under Your Seat*. Participants were required to indicate whether or not the event described had taken place, and any items they had doubts about could be left blank. The events were presented in a different order than in the video. Of the total items, 115 took place in the short film, while 40 did not. The response scale was dichotomous (Yes/No). The number of correct responses was converted into a scale of zero to ten: correct responses to all 155 items gave a score of ten, while incorrect responses resulted in a proportional drop in score. This questionnaire is included as supplementary material (S3 Questionnaire).
- **Spanish adaptation of Coleman and Borman’s (2000) scale of citizenship performance behaviours** [6]: originally composed of 27 items, 20 were selected for this study. Seven items were excluded from the scale because they represented citizenship behaviours that were not performed by the actors in the videos. These items were excluded because the behaviours recorded in them are absent from the videos. The response scale ranged from 1 to 7 with three anchors: Not at all characteristic, Characteristic and More characteristic than of anyone else, and was completed using a computer application. The reliability of the original scale is high (α = 0.96). Moreover, it produced a single (unidimensional) or several (multidimensional) measures of citizenship performance. In this work, we obtained a unidimensional measure of citizenship performance, and the reliability of the scale was 0.87.
- **Task performance assessment scale**: this tool was composed of 14 items based on the task inventory of a previous job analysis [44]. The participants used a computer application to evaluate the quality and frequency with which the main characters in each video performed the tasks associated with their jobs [45]. First, for each task, three behavioural descriptions appeared on screen. These descriptions represented different performance levels, though no explanation was given as to which performance level each one corresponded (Excellent, Good, Improvable). These response alternatives appeared randomly in a different position for each item. That is, the responses were not always displayed from deficient to excellent performance, or vice versa. Subsequently, when the level of performance was selected, three new alternatives were displayed, allowing the participant to report on the frequency with which the employee performed each task according to the level of quality previously indicated. Therefore, the response scale of this tool ranged from 1 to 9.

### Procedure

Participants were recruited on different days and from different class groups. Participants registered on various lists according to their availability, as the training programmes were held at different times. In each group the order of presentation of the two versions of the stimulus video was counterbalanced, so that half of each group assessed version 1 in the pre-test, version 2 in the post-test, and version 1 in the follow-up, while the other half began the procedure with version 2.

For the pre-test measure, participants were required to first complete both questionnaires on knowledge of performance and biases in the appraisal. Second, the short film *Life Vest Under Your Seat* was screened and participants completed the observational accuracy checklist. Third, the corresponding version of the video of the employee being assessed was shown. Participants could take notes during the presentation. Fourth, using a computer application, participants assessed citizenship and task performance of the main character.

All the groups, except control group, received training from a psychologist qualified in Psychology of Work and Organizations and with teaching experience. Training followed a participative methodology and was carried out in groups of seven to nine. Depending on the group, training lasted from two to thirteen hours. All groups completed the post-training measure (knowledge of performance and biases, observational accuracy, citizenship and task performance) four days after the pre-test measure. Finally, follow-up was done a month later by applying the same protocol as in previous moment measures.

**Fig 1:**
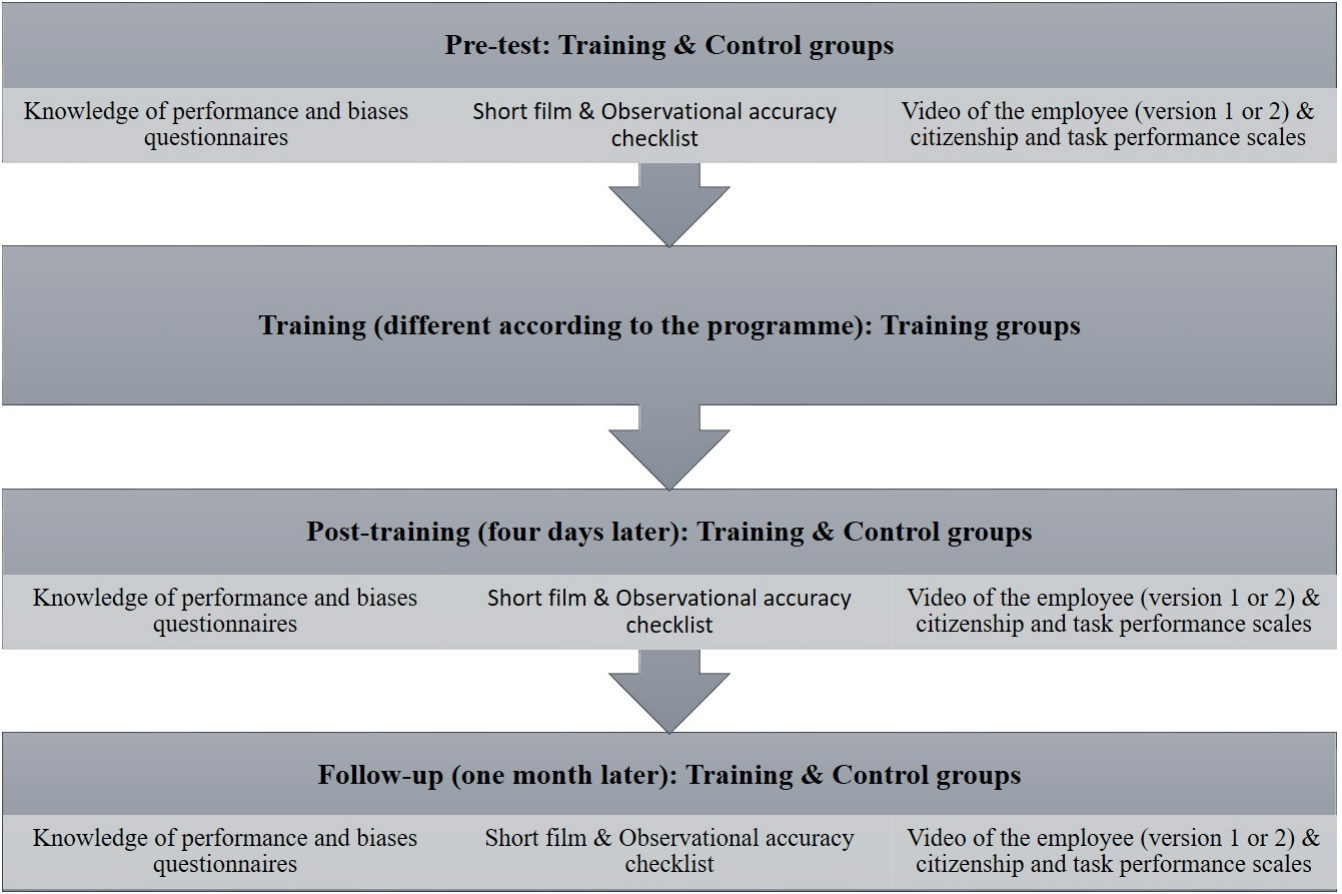
Phases of the procedure in studies 1 and 2.

The control group used the same instruments as the groups in training, in the same order and in the same period. The only difference with the other groups was that the control group received no training. Fig 1 gives the different phases of the procedure.

Participation in this study was voluntary and consented. Participation at all stages of the research was rewarded by a small increment in the final marks of a subject, once passed, of a second-year undergraduate degree, depending on the number of hours’ participation.

At the same time as student training was taking place, two versions of the video were shown to a group of three experts in Psychology of Work and Organizations with research and professional experience in performance assessment. Assessment of the citizenship and task performance of the employees featuring in both videos was obtained following the Delphi method. In the first stage, the three experts received an email containing the description of the fictitious employee’s job performance, along with several questionnaires for them to appraise the task and citizenship performance of this employee. The experts were given a week to return their scores. In the second stage, the researchers reduced the response scale anchors used in the performance assessment scales, only keeping the alternatives chosen most frequently by the group of experts. The evaluation questionnaires with modified response scales were resent to the experts, who were again given a week to assess the performance of the fictitious employee. This time they were also asked to explain and justify their responses. In the third stage, the questionnaires were rearranged to include only the most voted options on the response scale, as well as a summary of the experts’ most important comments on the character's performance. The experts then used these questionnaires as a basis to discuss the appropriate scoring of each item, subsequently reaching an agreement on the fictitious employee’s task and citizenship performance. This assessment was reached by consensus among the experts and was used as a criterion to evaluate the goodness-of-fit of participants’ assessments, in the understanding that assessments will be better the closer they are to those done by experts.

### Ethics statement

At the time of participant recruitment, because the study involved no risk to participants, informed consent was given verbally. Participants were clearly informed that participation was voluntary. When they came to take part in the pre-test checks, they gave their written consent. The study did not include minors. The University of La Laguna Ethics Committee in Tenerife, Spain (ULLECT) approved this study.

### Data and analysis

The database of this first study can be consulted at http://doi.org/10.3886/E109701V1. Data analysis was performed using IBM SPSS Statistics software, version 21.

## Results and discussion

First, typical scores and multivariate outliers with the Mahalanobis distance were used to analyze normality; three cases with atypical values were eliminated. The remaining analyses were made with 82 valid cases.

Second, we checked for differences in performance appraisals depending on the order of presentation of both versions of the video. Only one significant interaction of the Order of presentation with the Moment measure was obtained in citizenship performance appraisal (*F*(2, 79) = 15.24; *p* < .001; *η*^2^ = .28), so that the citizenship performance of the main character was valued more positively in the pre-training measure (Version 1 *M* = 3,69; Version 2 *M* = 2,94) (*t* = 4.68, *p* < .001) and follow-up (Version 1 *M* = 3,55; Version 2 *M* = 3,19) (*t* = 2.67, *p* < .01) in version 1 than in version 2 of the video. Third, groups were analyzed for differences in pre-test scores in each dependent variable, with only one significant difference being found in observational accuracy (*F*(4, 77) = 2.95; *p* < .05; *η*^2^ = .13), although a posteriori analysis with the Scheffé test did not reveal differences between groups.

Fourth, a one-way repeated measures multivariate analysis of variance (MANOVA) was undertaken using a between-group independent variable, Type of training, and the within-group variable, which corresponds to three moment measures for each dependent variable. Table 1 shows the descriptive statistics of the dependent variables in each group and moment measure. The experts’ scores for the fictitious employee’s performance were 4.9 (scale, 1–9) for task performance appraisal and 3.5 (scale, 1–7) for citizenship performance appraisal.

**Table 1:** Descriptive statistics of the dependent variables in each training group and moment measure (students).

		Moment measure						
		Pre-training		Post-training		Follow-Up		
		*M*	*SD*	*M*	*SD*	*M*		*SD*
Knowledge of performance and its dimensions	G1	5.6	2.8	9.7	0.8	8.6		1.9
	G2	5	2.1	7.5	2	7.3		2.1
	G3	4.9	2.8	6.9	2.6	7.0		1.8
	G4	6.4	2.0	9.6	1.0	9.5		1.1
	G5	5.8	1.5	6.7	2.1	6.9		1.9
Knowledge of biases in performance assessment	G1	3.1	1.8	3.2	1.6	4	2.6	
	G2	3.0	2.4	4.0	2.1	4.4	2.9	
	G3	2.7	1.6	7.7	1.7	7.4	1.4	
	G4	4.5	2.4	7.5	2.1	8.2	1.4	
	G5	3.5	2.1	4	2.2	4.3	2	
Observational accuracy	G1	4.5	1.0	5.4	1.6	6.2	0.8	
	G2	5.0	0.6	6.0	0.8	6.4	0.6	
	G3	4.1	0.9	5.6	0.8	6.3	0.6	
	G4	4.8	0.7	6.1	0.6	6.4	0.5	
	G5	4.8	0.7	5.9	1.1	6.6	0.7	
Task performance appraisal	G1	5.5	0.7	5.1	0.4	5.1	0.2	
	G2	5.8	0.4	5.7	0.5	5.8	0.6	
	G3	5.7	0.4	5.7	0.5	5.7	0.5	
	G4	5.4	0.5	5.0	0.5	5.1	0.4	
	G5	5.8	0.7	5.7	0.5	5.7	0.7	
	
Citizenship performance appraisal	G1	3.1	0.6	3.1	0.4	3.2	0.5	
	G2	3.1	0.6	3.2	0.7	3.4	0.5	
	G3	3.3	0.4	3.3	0.4	3.3	0.6	
	G4	3.1	0.9	3.1	0.3	3.1	0.4	
	G5	4.1	0.9	3.7	0.7	4.0	0.7	
	
Distance task performance appraisal	G1	0.6	0.7	0.4	0.3	0.2	0.2	
	G2	0.9	0.4	0.8	0.5	0.9	0.6	
	G3	0.7	0.4	0.8	0.5	0.8	0.5	
	G4	0.6	0.4	0.4	0.3	0.3	0.3	
	G5	0.9	0.8	0.8	0.5	0.8	0.7	
Distance citizenship performance appraisal	G1	0.6	0.4	0.5	0.4	0.5	0.4	
	G2	0.6	0.3	0.7	0.4	0.4	0.3	
	G3	0.4	0.2	0.3	0.3	0.5	0.4	
	G4	0.6	0.8	0.4	0.3	0.4	0.3	
	G5	0.7	0.8	0.6	0.4	0.7	0.6	

G1: Group training in Knowledge of dimensions and Frame-of-reference; G2: Group training in Observational accuracy; G3: Group training in Rater Error; G4: Group training in the previous three areas, Training_4_Programmes; G5: Control group.

### Effect on level of knowledge of performance and its dimensions

Significant main effects were obtained for Moment measure (*F*(2, 154) = 58.62; *p* < .001; *η*^2^ = .43; statistical power = 1.0) and Type of training (*F*(4, 77) = 7.51; *p* < .001; *η*^2^ = .28; statistical power = .99), as well as for interaction (*F*(8,154) = 2.35, *p* < .05; *η*^2^ = .11; statistical power = .88). A posteriori contrasts indicate that the groups trained in PDimT and FOR, and in Training_4_Programmes gain higher scores in knowledge of performance than the others, both during post-training and follow-up (*p* < .01).

### Effect on level of knowledge of biases in assessment

Main effects were obtained of Moment measures (*F*(2, 154) = 60.83; *p* < .001; *η*^2^ = .44; statistical power = 1.0) and Type of training (*F*(4, 77) = 12.31; *p* < .001; *η*^2^ = .39; statistical power = 1.0), as well as significant interaction (*F*(8, 154) = 10.61; *p*< .001; *η*^2^ = .35; statistical power = 1.0). A posteriori analyses showed that groups trained in Rater Error and in Training_4_Programmes scored higher in knowledge of biases in assessment than the other groups in the measure obtained after training and during follow-up (*p* < .001).

### Effect on observational accuracy

In this analysis, we used the Greenhouse-Geiser procedure to correct the degrees of freedom because Mauchly’s sphericity test was significant (*χ*^2^(2) = 13.29, *p* < .01). A significant main effect of Moment measure (*F*(2, 132) = 135.351; *p* < .001; *η*^2^ = .64; statistical power = 1.0) was obtained. A posteriori analyses showed that all the groups increased their observational accuracy in each new moment measure (*p* < .05).

### Effect on task performance assessment

The contrast was created using the Distance variable in relation to the expert group assessment, which was the absolute value resulting from subtracting the expert assessment from that made by each participant. Thus, assessments that were more similar to the expert assessment were considered more accurate.

In this case, as Mauchly’s sphericity test was significant (*χ*^2^(2) = 7.49, *p* < .05), we also used the Huynh-Feldt correction. Significant main effects were obtained for Moment measure (*F*(2, 151) = 3.92; *p* < .05; *η*^2^ = .05; statistical power = .70) and Type of training (*F*(4, 77) = 4.59; *p*< .01; *η*^2^ = .19; statistical power = .93), as well as for interaction (*F*(8, 154) = 2.13; *p* < .05; *η*^2^ = .10; statistical power = .83). A posteriori contrasts showed that the task performance appraisal carried out by groups trained in Dimensions and Frame-of-reference, and in the Training_4_Programmes were more similar to the expert version than the other groups.

### Effect on citizenship task performance assessment

No main effects or statistically significant interaction were found and subsequently there was no variation in the type of training given or in the different moments of measure of participants’ citizenship performance appraisals.

## Conclusions

The results confirm the first two hypotheses. Knowledge of performance and its dimensions, and of biases in assessment has increased in the groups that received specific training, either through an independent programme or through the Training_4_programmes. This study contributes interesting data on the usefulness of training programmes for increasing knowledge of the performance dimensions and biases that may arise during appraisal. The results also show the stability of the knowledge acquired over time, an aspect of training in Rater Error that has been specifically criticized [21]. The results did not corroborate hypothesis 1.3, in relation to observational accuracy, since participants in all the experimental groups, regardless of type of training and whether or not they had received any, were more accurate in each new moment measure.

The fourth hypothesis, which considered that the group trained in all four programmes would produce a task and citizenship performance appraisal closer to the expert judgement than the other groups, was partially confirmed. Moreover, task performance assessment revealed that the scores of both the group that received Training_4_Programmes and the group trained in FOR and PDimT were closer to those issued by the group of experts than the control group and other experimental groups. This result concurs with that of previous studies, where participants trained in the creation of a common frame-of-reference, either independently or combined with other types of training, gave more accurate appraisals than those who had received no training or minimal training [46,33,34,39,41,14,15,29,47,35,48,36,37,49,27,25,50,18,32,51,52,28,16,17,53,54]. The results of this study, unlike those obtained in other studies [23,7], show that training in Rater Error included in a combined programme does not affect appraisal accuracy. In citizenship performance assessment, none of the groups showed any improvement in assessment accuracy.

The results of this study must be interpreted bearing in mind that the sample is made up of students. The following research replicates this study with a sample of employees, considering only the training programmes that engender more effective assessment in relation to a group with no training.

## Second study

The objective of this study, using a sample of employees, is to test the effectiveness of the two training programmes that obtained the best results in the previous study: Knowledge of dimensions and Frame-of-reference (KdFOR), and Training_4_Programmes. The specific hypotheses proposed for this second study are:

*Hypothesis 2*.*1* (*H2*.*1*): groups receiving training score higher in general knowledge of performance than the control group.

*Hypothesis 2*.*2* (*H2*.*2*): groups trained in all four programme types will score higher in knowledge of appraisal biases than the other groups.

*Hypothesis 2*.*3* (*H2*.*3*): groups trained in all four programme types will identify more accurately the occurrence or non-occurrence of various events.

*Hypothesis 2*.*4* (*H2*.*4*): group trained in all four training programmes will produce a task and citizenship performance appraisal closer to the expert judgement than the other groups.

## Method

### Participants

G*Power: Statistical Power Analyses revealed that sample size should be 54 people, with a 95% confidence level and 5% margin of error with three groups and five dependent variables. Given the mortality rate of the sample, the sample consisted of 42 employees from different organizations, of whom 59.5% were women and 40.5% men. The average age was 43.52 years (range, 28–60 years). Of the participants, 92.85% had received a university education and the rest secondary schooling. Participants held positions of responsibility over other persons or positions where they were required to assess other employees. Following the International Standard Classification of Occupations (ISCO-88), 42.85% of the employees held management or middle management positions in administration and human resources, 54.76% were professionals or mid-level technicians (e.g., lawyers, advisors, nurses, psychologists, teachers), and 2.38% were office workers. Of the sample, 54.8% had some prior experience in performance appraisals.

### Design

We used the same design as in the first study: quasi-experimental, factorial-multivariable and longitudinal (repeated measures) analysis, obtaining for each employee three measures gathered at three different moments: before and after training, and a month after training (follow-up), which constituted the within-group variable: Moment measure. Three groups were created for the between-group variable, Type of training: 1) training in Knowledge of dimensions and Frame-of-reference (KdFOR) (*n* = 15), 2) Training_4_Programmes (*n* = 13), and 3) Control group (without training, *n* = 14). The dependent variables were the same.

### Tools and procedure

We used the same materials and tools outlined in the previous study. Participants were recruited from various public and private companies, and were organized into training and control groups, according to their availability. The procedure was the same as in the first study (Fig 1).

Employee participation in this study was voluntary and consented. By way of incentive, participants received some economic compensation at the end of each research stage, according to the time spent. In this study, the order of presentation of both versions of the video used as a stimulus for performance appraisal was not counterbalanced because of the difficulty in adjusting timetables to employee availability.

### Ethics statement

Because the study involved no risk to participants, informed consent was given verbally. A meeting was held to provide information about the research project: participation was voluntary and participants could leave at any time; the data collected would be used exclusively for research purposes; personal data protection was ensured; and participation signified that participants gave their consent to the use of the research data. The University of La Laguna Ethics Committee in Tenerife, Spain (ULLECT) approved this study.

### Data and Analysis

The database of this second study can be consulted at http://doi.org/10.3886/E109701V1. Data analysis was also performed using IBM SPSS Statistics software, version 21.

## Results and discussion

Firstly, we checked for the absence of univariate and multivariate outliers. Typical scores were used to test univariate outliers. Multivariate outliers were checked with Mahalanobis distance. No outliers were found. Secondly, to test the effect or previous experience in performance appraisal, MANOVA was undertaken using two between-group independent variables, Type of training and Previous experience in performance appraisal, and the within-group variable, Moment of measure, for each dependent variable. No main effects or statistically significant interaction were found. Thirdly, groups were analyzed for differences in pre-test scores in each dependent variable, with only one significant difference being found in knowledge of performance and its dimensions (*F*(2, 39) = 8.46; *p* < .01; *η*^2^ = .30). A posteriori analysis with the Scheffé test revealed that the control group shows a lower score for Knowledge of performance and its dimensions (*p* < .05) when compared with the group trained in Dimensions and Frame-of-reference. Table 2 shows the descriptive statistics of the dependent variables in each group and moment measure.

**Table 2:** Descriptive statistics of the dependent variables in each training group and moment measure (employees).

		Moment measure					
		Pre-training		Post-training		Follow-Up	
		*M*	*SD*	*M*	*SD*	*M*	*SD*
Knowledge of performance and its dimensions	G1	7.02	2.70	8.40	2.70	9.21	1.49
	G2	5.80	1.20	8.18	1.48	8.81	1.01
	G3	4.10	1.41	4.46	1.28	4.57	1.19
Knowledge of biases in performance assessment	G1	3.97	3.10	4.22	3.30	4.67	3.24
	G2	2.71	2.63	6.92	1.96	7.69	1.13
	G3	2.47	0.99	3.25	0.94	3.78	1.26
Observational accuracy	G1	4.80	0.79	5.64	0.63	6.05	0.87
	G2	4.52	0.87	5.78	0.52	6.23	0.47
	G3	4.18	0.90	5.72	0.85	6.14	0.72
Task performance appraisal	G1	5.6	1.0	5.3	0.7	5.0	0.9
	G2	5.6	0.4	5.3	0.3	4.9	0.3
	G3	6.0	0.7	5.8	0.5	5.7	0.7
	
Citizenship performance appraisal	G1	3.3	0.8	2.9	0.6	3.2	0.7
	G2	3.3	0.5	3.3	0.3	3.4	0.2
	G3	4.1	0.3	4.4	0.3	4.4	0.5
	
Distance task performance appraisal	G1	1.0	0.6	0.6	0.4	0.6	0.6
	G2	0.7	0.4	0.4	0.3	0.2	0.2
	G3	1.1	0.7	0.9	0.5	0.9	0.7
Distance citizenship performance appraisal	G1	0.7	0.4	0.7	0.5	0.6	0.5
	G2	0.4	0.3	0.3	0.3	0.2	0.1
	G3	0.6	0.3	0.9	0.3	0.9	0.5

G1: Group training in Knowledge of dimensions and Frame-of-reference; G2: Group training in the previous three areas, Training_4_Programmes; G3: Control group.

Fourthly, a one-way repeated measures multivariate analysis of variance (MANOVA) was undertaken using a between-group independent variable, Type of training, and the within-group variable, Moment of measure, for each dependent variable.

### Effect on level of knowledge of performance and its dimensions

Significant main effects were obtained for the Moment measure (*F*(2, 78) = 20.887; *p* < .001; *η*^2^ = .35; statistical power = 1.0) and Type of training (*F*(2, 39) = 33.92; *p* < .001; *η*^2^ = .64; statistical power = 1.0), as well as for interaction (*F*(4, 78) = 3.377; *p* < .05; *η*^2^ = .15; statistical power = .83). A posteriori contrasts indicate that the two trained groups gained higher scores in Knowledge of performance than the control group (*p* < .05), although these differences already existed in the first moment measure (pre-training) of the group trained in Dimensions of performance and Frame-of-reference.

### Effect on level of knowledge of biases in assessment

As Mauchly’s sphericity test was significant (*χ*^2^(2) = 7.950; *p* < .05), we also used the Greenhouse-Geisser procedure to correct the degrees of freedom. Significant main effects were obtained for Moment measure (*F*(2, 66) = 26.61; *p* < .001; *η*^2^ = .41; statistical power = 1.0) and Type of training (*F*(2, 39) = 6,071; *p* < .001; *η*^2^ = .24; statistical power = .86), as well as for interaction (*F*(3, 66) = 9,714; *p* < .001; *η*^2^ = .33; statistical power = .99). A posteriori analysis showed that the group that received Training_4_Programmes scored higher in Knowledge of biases in assessment than the other groups (*p* < .01).

### Effect on observational accuracy

As Mauchly’s sphericity test was significant (*χ*^2^(2) = 12.06; *p* < .01), we used the Huynh-Feldt correction. A significant main effect was obtained for Moment measure (*F*(2, 67) = 151.709; *p* < .001; *η*^2^ = .80; statistical power = 1.0) and interaction (*F*(3, 67) = 3.110; *p* < .05; *η*^2^ = .14; statistical power = .74). A posteriori analysis showed no significant differences between the groups for the three moment measures.

### Effect on task performance assessment

As before, we calculated the effectiveness of the performance appraisals using distance in relation to the expert group assessment. Main effects were obtained for Moment measure (*F*(2, 78) = 12.221; *p* < .001; *η*^*2*^ = .24; statistical power = .99), and Type of training (*F*(2, 39) = 4.860; *p* < .05; *η*^2^ = .20; statistical power = .77). A posteriori contrast between the various moment measures gave significant differences between the pre- and post-training measures (*p* < .05), and follow-up (*p* < .05). A posteriori contrast with the Scheffé test in the Type of training variable showed that the group that received Training_4_Programmes produced more accurate assessments than the control group (*p* < .05).

### Effect on citizenship task performance assessment

A significant main effect was obtained for the variable Moment Training (*F*(2, 39) = 8.374; *p* < .01; *η*^2^ = .30; statistical power = .95) and interaction (*F*(4, 78) = 3.717; *p* < .01; *η*^2^ = .16; statistical power = .87). A posteriori contrasts indicate that the group that received Training_4_Programmes issued a more accurate citizenship performance appraisal than the other two groups (*p* < .05).

## Conclusions

The results of this second study concur with those previously obtained in this work and with those of other authors: trained employees show a higher level of knowledge in the post-training and follow-up measure [39,27,16]. As considered in hypothesis 2.1, trained groups score higher in knowledge of performance than the control group. Although this difference was already significant in the moment measure before training, especially for the group trained in Dimensions and Frame-of-reference, descriptive statistics show that the higher score in knowledge is greater for the two trained groups. Likewise, the group that received Training_4_programmes improved knowledge of biases in assessment, in line with hypothesis 2.2. For observational accuracy, contrary to hypothesis 2.3, once again all the groups showed greater accuracy in each new moment measure.

The results confirm hypothesis 2.4 because the group trained in all four programmes produced a task and citizenship performance appraisal closer to the one issued by the expert rater group than the control group.

## General discussion

The aim of this study was to analyze how the type of theoretical and practical training influences performance appraisal. The first study compared performance appraisal by students who were trained in four types of programmes (Knowledge of dimensions and Frame-of-reference, KdFOR; Observational accuracy; Rater Error Training, RET; and Training_4_programmes) and that conducted by those who received no training. The second study tested the effectiveness among employees of the two training programmes: Knowledge of dimensions and Frame-of-reference, and the Training_4_Programmes, which gave better results in the first study.

Both studies found that training increases knowledge of performance and its dimensions, and of biases in assessment, as was posited in hypotheses 1.1, 2.1 and 1.2, 2.2. Thus, for students and employees alike, the training programmes facilitate the acquisition of knowledge about bias identification and performance dimensions, as well as the development of a framework-of-reference shared by the raters. This knowledge can improve performance appraisal accuracy. To this end, data on the effectiveness of training programmes are provided, an issue highlighted by some authors as one of the aspects that requires further study in this field [55].

In relation to the third hypothesis (1.3 and 2.3), we analyzed the rating accuracy of participants in the various training groups when deciding on the occurrence or non-occurrence of several events from a list about a short film. This kind of analysis is similar to one previously conducted by other researchers who attempted to assess whether suitable or specific training can increase rater observational accuracy [33,27,32,28,16,26,19]. Likewise, most of these authors indicated that, although training does not improve performance assessment accuracy, it is beneficial in terms of recognition and recall. In both studies, the hypothesis raised was rejected. All participants, trained or otherwise, were more accurate at each new assessment moment. That is, the score of all participants improved in the post-training moment measure, in comparison with the pre-training measure, which was lower than that obtained during follow-up. A plausible explanation can be the learning associated with the task, along with repeated exposure—three times—to the list of events and behaviours, and to the short film.

By contrasting with the fourth hypothesis (1.4 and 2.4), we have attempted to decide whether task and citizenship performance appraisal varies according to the type of training received. Several studies have analyzed the effectiveness of the various types of rater training along these lines [56,14,57,35,27,50,32,13,52,16,53,54,7]. Likewise, in order to evaluate the improvement of assessment accuracy, most studies [58,14,15,18,16,17,54,19] have used Cronbach’s indices [11] or Borman’s distance accuracy index [12]. In this study, we chose Borman’s index [12], using the scores given by a group of experts as a reference measure to evaluate appraisal effectiveness.

The results of the first study showed that raters who received training in frame-of-reference or the Training_4_Programmes produced a more accurate performance appraisal, in line with other works [34,41,14,15,40,35,36,37,49,27,18,32,51,17,53,54]. For citizenship performance appraisal, however, training did not improve student accuracy, as in the study by Sulsky et al. [53]. This result may be due to the difficulty of capturing citizenship performance in a video, thereby making assessment difficult, especially for students. However, the results of the second study show greater accuracy in both task and citizenship performance assessment when employees receive training in the Training_4_programmes.

The results allow us to draw a series of conclusions. First, that the group trained in Dimensions and Frame-of-reference excelled compared with others is in line with the results of other authors, showing once again the effectiveness of this type of programme [14,56,35,13,52,16,53,54]. Second, that the increased accuracy of appraisal by the Training_4_Programmes group goes against the results obtained by Noonan and Sulsky [27], who point out that the combined use of several types of training does not lead to a significant increase in effectiveness, beyond the improvement obtained from implementing the programmes separately. However, it supports the work of Eppich et al. [41], who achieved considerable improvement in rater accuracy by combining all types of training strategies, despite a small sample size. Moreover, a positive aspect of the studies presented in this paper is that, unlike that of Eppich et al. [41], appraisal accuracy is measured by using true scores from a group of experts. Third, Sulsky et al. [53] highlight the importance of citizenship behaviours as an essential part of employees’ daily work. An important contribution of this study is the inclusion of citizenship performance assessment as a trainable aspect, since only very few studies have used training in citizenship performance appraisal and have shown the effectiveness of that training. Future research should continue to explore how the accuracy of citizenship performance appraisal can be improved and why training is sometimes effective and sometimes not, as shown by these results.

Another contribution of this study is the comparison of all the training programmes categorized by Woehr and Huffcutt [7], as well as their combination and comparison with an untrained group, thereby facilitating the evaluation of the effectiveness of various components covered in each training programme.

These findings are not exempt of certain limitations when the results are generalized. On the one hand, the sample used in the first study was composed of students whose perspective may be distanced from the reality of the world of work. Nevertheless, although performance assessment is associated with employees, it is routine practice to use students in research focusing on performance rater training. Laboratory situations are presented with a fictitious evaluation task in which students are required to play the role of the rater [27]. In the second study, the sample was made up of employees, whose limited availability curbed the counterbalancing of the order of presentation of the videos used as a stimulus for performance assessment and random assignment to experimental groups. Moreover, this sample contained a high percentage of participants with university studies. Using raters with a different level of study may give different results.

Moreover, it would have been advisable to have a larger group size. However, these types of longitudinal studies that require considerable time involvement from participants are associated with difficulties in recruiting and maintaining the sample. Despite efforts to increase sample size, it was not possible, and the power of the results was analyzed a posteriori, revealing an adequate value in most cases.

On the other hand, another improvable aspect is the use of videos as a base material for carrying out performance appraisal. In this regard, Noonan and Sulsky [27] were the first authors to study the effectiveness of rater training (FOR and BOT) in the applied field, since, until then, all studies had used laboratory situations with a fictitious assessment task. Their results show that in applied fields and with assessments of real employees training also improves assessment effectiveness. However, in order to claim that the effect of training is greater or more easily assimilated when real employees are rated, this procedure should be compared with a trained group rating fictitious employees. That said, in future, it would be interesting to implement programmes that have excelled in rater training in one or several organizations, so that pre- and post-training, and follow-up measures of performance appraisal are of real employees, with whom raters interact on a daily basis.

Finally, the contribution of this study to the field of rater training programmes is worth noting. Students in the experimental group in Training_4_Programmes and participants in the Frame-of-reference group excelled, gaining the best scores in both knowledge tests and a more accurate task performance appraisal. This finding is a further step ahead in rater training, since a training programme that includes features of all kinds of programmes can be equally effective as Frame-of-reference training, pinpointed in numerous studies as offering greater accuracy [41,14,56,54]. Likewise, when applied to students, the longer combined programme does not necessarily give better results than a shorter training programme; the cost of implementation would therefore not recommend its use. However, when employees receive training, the combined training programme has been shown to be more effective in both types of performance appraisal. Therefore, when the aim of the organization is accurate appraisal in both task and citizenship performance, Training_4_Programmes is more appropriate.

## Supporting information

S1 Appendix Summary of objectives, contents, and length of training programmes. (PDF)

S1 Questionnaire Questionnaire on knowledge of performance and its dimensions. (PDF)

S2 Questionnaire Questionnaire on knowledge of biases in performance assessment. (PDF)

S3 Questionnaire Checklist of observational accuracy. (PDF)
