# Supplementary material for: Does effectiveness in performance appraisal improve with rater training?
Source: PLoS One. 2019 Sep 19;14(9):e0222694. doi: 10.1371/journal.pone.0222694 (PMC6752840; doi:10.1371/journal.pone.0222694)
Supplement: S1 Appendix — (PDF) [file pone.0222694.s001.pdf]

S1 Appendix. Summary of objectives, contents, and length of training programmes.

PROGRAMME	OBJECTIVE	TIME	CONTENT
Knowledge of dimensions and Frame-of-reference	Discovering what performance appraisal is and what it consists in, its multidimensionality, and common standards for evaluation	2 hours	**Unit 1**<br>Theoretical explanation of concept and performance dimensions, and frame-of-reference.<br>Practical exercise 1: Identification and creation of job tasks.<br>Practical exercise 2: Distinction between task and citizenship performance.<br>Practical exercise 3: Search for group consensus on task and citizenship performance dimensions.<br>Solution of questions about performance.<br>Summary of the unit.
Observational accuracy	Improvement of rater observational accuracy	2 hours	**Unit 1**<br>Theoretical explanation of the importance of identifying data relevant to employee performance.<br>Practical exercise: Training in observation and recall of behaviours with videos.<br>Summary of the unit.
Rater Error	Reducing the occurrence and effect of biases in assessment based on definitions of standard errors and their likely causes; analysis of examples of errors and the strategies that can be used to avoid them	8 hours	**Unit 1. Bias of similarity and contrast.**<br>Activity of discovery: Exercise of performance appraisal of employees with whom the rater has a high and low level of identification, analysis of the differences between assessments of both, theoretical conclusion.<br>Practical exercise: Two raters' analysis of performance appraisals of two training videos which show two administrative employees (one with good performance and the other with improvable performance), results analysis, and conclusions.<br>Solution of questions about these biases.<br>Summary of the unit.<br>**Unit 2. Biases of primacy, recency, and negativity.**<br>Activity of discovery: Memorization exercise and recall of series of elements, results analysis, theoretical conclusion.<br>Practical exercise 1: Tasks and citizenship behaviours recalled from a performance diary, results analysis, and conclusions.<br>Practical exercise 2: Memorization and recall of adjectives, results analysis, and conclusions.

			Practical exercise 3: Role plays and assessment of behaviours, results analysis, and conclusions.<br>Solution of questions about these biases.<br>Summary of the unit.<br>**Unit 3. Biases of first impression, halo effect, and spillover.**<br>Activity of discovery: Recall exercise of tasks and behaviours of the characters in an incomplete comic, inclusion of final cartoons, reflection and debate, theoretical conclusion.<br>Practical exercise 1: Analysis of photographs of employees, account with a description of conduct, introduction of new information, results analysis, and conclusions.<br>Practical exercise 2: Account of images and sayings by historical characters, results analysis, and conclusions.<br>Practical exercise 3: Presentation of performance appraisals made by two supervisors of two employees, from two training videos, performance appraisal of the same employees, results analysis, and conclusions.<br>Solution of questions about these biases.<br>Summary of the unit.<br>**Unit 4. Biases of leniency, central tendency, and severity.**<br>Activity of discovery: Exercise on conceptions and thoughts of the teaching staff, reflection, debate, and theoretical conclusion.<br>Practical exercise: Presentation of different rater roles, performance appraisals in different roles, assessment in the appropriate role, results analysis, and conclusions.<br>Solution of questions about these biases.<br>Summary of the unit.<br>**Unit 5. Biases of attribution and stereotypes.**<br>Activity of discovery: Exercise of association between professions and nationalities of fictitious employees, subsequent association with a description of these employees, performance appraisal of the employees, reflection, debate, and theoretical conclusion.<br>Practical exercise: Presentation of a training video and videos of everyday situations, individual analysis, reflection and group debate, results analysis, and conclusions.<br>Solution of questions about these biases.<br>Summary of the unit.
Training_4_Programmes	The previous three	12 hours	All the previous
