# Supplementary material for: Does effectiveness in performance appraisal improve with rater training?
Source: PLoS One. 2019 Sep 19;14(9):e0222694. doi: 10.1371/journal.pone.0222694 (PMC6752840; doi:10.1371/journal.pone.0222694)
Supplement: S1 Questionnaire — (PDF) [file pone.0222694.s002.pdf]

S1 Questionnaire. Questionnaire on knowledge of performance and its dimensions

Datos personales (EN MAYÚSCULAS)

Apellidos (en mayúsculas):
Nombre:
ID:
Fecha:

Instrucciones

A continuación se presentan **11 preguntas** sobre el desempeño laboral, sus dimensiones y su evaluación.

Por favor, lea detenidamente cada pregunta y decida, según su criterio y conocimiento, si esa afirmación es VERDADERA O FALSA. ***Cada error que cometa supone el descuento de una respuesta correcta en la puntuación final***, de manera que, si no está 100% seguro de su respuesta, deje esa pregunta en blanco y pase a la siguiente.

Recuerde que este cuestionario es parte de la investigación en la que está participando y su único objetivo es analizar los conocimientos que adquirirán con el programa de formación. Por favor, lea con atención las preguntas antes de responderlas.

		V	F
1	El desempeño de tarea se refiere a cuántas tareas realiza el trabajador cada día.		
2	Un trabajador tendrá un buen desempeño de tarea cuando realice adecuadamente las tareas asignadas a su puesto.		
3	El desempeño cívico se refiere a comportamientos que, aun no estando establecidos formalmente en los puestos, facilitan el desarrollo del trabajo.		
4	El desempeño cívico se refiere a las tareas que debe desempeñar el trabajador en su puesto.		
5	Evaluar el desempeño de tarea consiste en valorar la calidad y la frecuencia con la que un empleado realiza las actividades asociadas a su puesto.		
6	La voluntariedad de un trabajador para realizar tareas adicionales en su puesto es una conducta de desempeño cívico.		
7	Ayudar a otros compañeros es un comportamiento que define el buen desempeño de tarea de un trabajador.		
8	En las evaluaciones de desempeño, es importante que los evaluadores acuerden y compartan entre ellos las descripciones de los puestos a evaluar.		
9	No es necesario tener un conocimiento previo de las tareas y conductas requeridas por un puesto de trabajo para evaluar el desempeño del trabajador que la realiza.		
10	Todos los evaluadores de una organización deben compartir un marco de referencia común.		
11	Un buen evaluador puede evaluar a un trabajador sin definir anteriormente los niveles de calidad de realización de cada una de las tareas.		

Muchas gracias por su colaboración.
