# Supplementary material for: Does effectiveness in performance appraisal improve with rater training?
Source: PLoS One. 2019 Sep 19;14(9):e0222694. doi: 10.1371/journal.pone.0222694 (PMC6752840; doi:10.1371/journal.pone.0222694)
Supplement: S2 Questionnaire — (PDF) [file pone.0222694.s003.pdf]

S2 Questionnaire. Questionnaire on knowledge of biases in performance assessment

Datos personales (EN MAYÚSCULAS)

Apellidos (en mayúsculas):
Nombre:
ID:
Fecha:

Instrucciones

A continuación se presentan **21 preguntas** sobre los errores más comunes que comenten los evaluadores al valorar a los trabajadores bajo su supervisión.

Por favor, lea detenidamente cada pregunta y decida, según su criterio y conocimiento, si esa afirmación es VERDADERA O FALSA. ***Cada error que cometa supone el descuento de una respuesta correcta en la puntuación final***, de manera que, si no está 100% seguro de su respuesta, deje esa pregunta en blanco y pase a la siguiente.

Recuerde que este cuestionario es parte de la investigación en la que está participando y su único objetivo es analizar los conocimientos que adquirirán con el programa de formación. Por favor, lea con atención las preguntas antes de responderlas.

		V	F
1	El sesgo de similitud produce evaluaciones más positivas de los trabajadores más parecidos al evaluador.		
2	El sesgo de similitud se refiere a que los evaluadores sólo deben evaluar a trabajadores que ocupen puestos similares al suyo.		
3	Cuando un evaluador evalúa a los trabajadores comparándolos entre sí incurre en el sesgo de contraste.		
4	El sesgo de contraste se refiere a la tendencia a evaluar el desempeño del trabajador en relación con unos criterios establecidos previamente.		
5	El sesgo de primacía se produce cuando la evaluación del desempeño se basa en la información recopilada en las fases iniciales del periodo a evaluar.		
6	El sesgo de recencia se produce cuando la evaluación del desempeño se basa en la información recolectada en las últimas fases del periodo a evaluar.		
7	El sesgo de negatividad se produce cuando los evaluadores dan mayor peso a la información negativa que a la positiva y neutral.		
8	El sesgo de negatividad se refiere a cuando el evaluador valora negativamente todas las conductas del trabajador.		
9	El sesgo de primera impresión se produce cuando el evaluador valora a un trabajador en función de la imagen que se formó del mismo al conocerle y no en base a su comportamiento posterior.		
10	Cuando un evaluador evalúa a un trabajador considerando una única cualidad del trabajador está incurriendo en el efecto halo.		
11	El efecto de halo se refiere a la huella mnémica que deja un trabajador al realizar su trabajo y que influye en la evaluación.		
12	El efecto de spillover se produce cuando una evaluación en curso se ve influida por los resultados de una evaluación previa.		
13	El sesgo de benevolencia se produce cuando los evaluadores asignan puntuaciones elevadas a la mayoría o a todos los empleados.		
14	El sesgo de benevolencia se produce cuando varios evaluadores son benevolentes y asignan puntuaciones elevadas a un trabajador.		
15	El sesgo de tendencia central se produce cuando los evaluadores usan, solamente, los puntos medios de la escala, evitando utilizar los extremos.		
16	El sesgo de severidad se produce cuando los evaluadores asignan puntuaciones bajas a la mayoría o a todos los empleados.		
17	El sesgo de severidad se produce cuando se puntúa especialmente mal a un trabajador al haber cometido un error.		
18	El sesgo de atribución se produce cuando un evaluador atribuye únicamente a las características personales del empleado su bajo desempeño laboral.		
19	El sesgo de atribución se produce cuando un evaluador atribuye a la mala suerte el bajo desempeño del trabajador.		

20	Cuando un trabajador es evaluado atendiendo a las características atribuidas a un grupo al que puede pertenecer, el evaluador estará usando un estereotipo.		
21	Es útil para realizar una buena evaluación tener en cuenta cómo son las personas que suelen ocupar los puestos a su cargo.		

Muchas gracias por su colaboración.
