# Supplementary material for: Does effectiveness in performance appraisal improve with rater training?
Source: PLoS One. 2019 Sep 19;14(9):e0222694. doi: 10.1371/journal.pone.0222694 (PMC6752840; doi:10.1371/journal.pone.0222694)
Supplement: S3 Questionnaire — (PDF) [file pone.0222694.s004.pdf]

S3 Questionnaire. Checklist of observational accuracy

Datos personales (EN MAYÚSCULAS)

Apellidos (en mayúsculas):
Nombre:
ID:
Fecha:

Instrucciones

A continuación se presenta un listado de 155 sucesos y acciones que pueden haber sucedido o no en el video.

Por favor, lea detenidamente cada pregunta y pregúntese *si ese acontecimiento ocurre*.

Marque con una cruz en la casilla **SÍ** si usted cree que dicho suceso o acciones ha sucedido en el video; marque la casilla **NO** si usted cree que no ha sucedido; o deje la casilla en **blanco** si no está seguro de si ha sucedido o no dicha acción.

Recuerde que este cuestionario es parte de la investigación en la que está participando y su único objetivo es analizar los conocimientos que adquirirán con el programa de formación.

Por favor, **lea con atención las preguntas antes de responderlas.**

		SI	NO
1	El protagonista se llama Mauro.		
2	Mauro tiene barba, cejas pobladas y ojos oscuros.		
3	Las cortinas de separación de secciones son blancas.		
4	Mauro recorre todo el avión persiguiendo a Luisa para hablar con ella.		
5	La sobrecargo se llama Luisa.		
6	El niño está jugando con el teléfono móvil.		
7	El comandante informa que el responsable del imprevisto se hará cargo de los billetes y de todos los gastos ocasionados.		
8	La azafata le sirve una bebida al hombre que inicia la historia del secuestro.		
9	Los asientos de primera clase son marrones-beige.		
10	El azafato lleva chaleco.		
11	El comandante es calvo.		
12	Luisa le pone dos tapones de algodón en la nariz al niño para que deje se sangrar.		
13	Mauro presiona y chantajea al comandante y copiloto con la difusión por televisión, periódicos, radio e internet de la noticia del secuestro (sea o no real).		
14	Luisa le sirve el menú de pasta de Mauro.		
15	Los pasajeros levantan su mano con el teléfono móvil en ella para entregárselo a la azafata.		
16	El comandante informa que la aerolínea se hará cargo de la devolución de los billetes y de los gastos ocasionados por regresar al aeropuerto de origen, Madrid-Barajas.		
17	Mauro va al baño del avión.		
18	El hombre sentado en primera clase tiene barba, lleva corbata y chaqueta.		
19	Un hombre con perilla y camisa rojo-teja de manga corta informa del secuestro al protagonista.		
20	En primera clase, cada asiento tiene su propia pantalla.		
21	El azafato lleva camisa blanca y corbata.		
22	Mauro se une al grupo que está decidiendo si actuar o no.		
23	Mauro usa una cerilla para encender el cigarro en el baño.		
24	Después del “accidente” del niño, Luisa se marcha sin mediar palabra con Mauro.		
25	La madre del niño lo regaña por patear el asiento del viajero de delante (Mauro).		
26	La azafata y el azafato están en la puerta de la cabina del comandante.		
27	Las azafatas llevan zapatos de tacón.		

28	De la mayoría de los armarios superiores para guardar el equipaje de mano, cuelgan las asas de bolsos y maletas de los pasajeros; así como, ropa de abrigo.		
29	Mauro trata de aclarar con Luisa lo sucedido durante el vuelo (comida y niño).		
30	Luisa recorre todo el pasillo del avión sin atender las llamadas de los pasajeros.		
31	El niño llama a su madre al recibir el golpe.		
32	Al presentarse, Germán da dos besos a Mauro.		
33	La noticia del secuestro llega hasta los pasajeros de primera clase.		
34	El azafato se marcha del lugar donde transcurre la conversación entre Mauro y Luisa.		
35	El niño es rubio de pelo medio-largo, lleva polo blanco y pantalón corto azul marino.		
36	La consola de información de la ruta se modifica, el avión apunta de nuevo rumbo a España.		
37	Luisa tiene un ojo morado y el labio hinchado.		
38	El polo del niño permanece manchado de sangre todo el vuelo.		
39	Una pasajera usa una almohada con funda para dormir.		
40	Tras el incidente del niño, Mauro está leyendo un libro.		
41	Mauro informa al comandante y al copiloto que todos los pasajeros piensan que ellos, junto con la tripulación, han secuestrado el avión.		
42	Cuando Mauro se cambia de asiento, la mujer de al lado está durmiendo.		
43	Luisa entra en el baño porque salta la alarma del sistema de detección de humo.		
44	La tripulación (sobrecargo, azafata y azafato) ajenos a la noticia del secuestro, están hablando y riéndose entre ellos.		
45	Mauro lleva un reloj en su mano izquierda.		
46	Cuando Mauro está en el baño, se ve una pulsera en su muñeca derecha.		
47	Antes de que la azafata descubra el motín, las pantallas muestran la ruta de vuelo.		
48	Luisa invita al niño y a su madre a pasar a primera clase.		
49	Mauro exige hablar con el comandante.		
50	La noticia del secuestro comienza a extenderse por todos los pasajeros del avión.		
51	Luisa y la azafata sirven la comida a todos los pasajeros del avión.		
52	Las azafatas llevan un pañuelo al cuello trabado con un alfiler.		
53	Los asientos del avión son azules con el emblema de la compañía en amarillo.		
54	Mauro patea con los dos pies el asiento del niño desde atrás.		

55	Mauro está durmiendo cuando el niño empieza a patear su asiento.		
56	Al tratar de impedir que la lleven a la cabina, a Luisa se le ve la ropa interior.		
57	La azafata no lleva chaleco como Luisa.		
58	Uno de los pasajeros confabuladores, el que viste camisa roja de manga corta, (German) se suena varias veces antes de tomar asiento.		
59	El comandante informa que la temperatura exterior en Miami es de 35º.		
60	Mauro enciende un cigarro dentro del baño del avión mientras está sentado en el inodoro.		
61	El comandante tiene barba.		
62	Hay extintores en las paredes de diferentes puntos del avión.		
63	Mauro se muestra nervioso, tras conocer que regresan a Madrid.		
64	Cuando Luisa localiza a los pasajeros, éstos la están amenazando con diversos objetos (muleta, tenedor, extintor, cafetera, cazo, pinzas,…).		
65	Al entrar la azafata en la zona turista, los pasajeros dejan de hablar y la miran aterrorizados.		
66	La azafata coloca y acomoda la almohada del pasajero que comenzó la historia del secuestro.		
67	Luisa recoge todos los teléfonos móviles.		
68	Tras la noticia del secuestro, Mauro enciende su móvil para llamar por teléfono.		
69	Un pasajero de traje y barba comienza a crear el pánico informando de que una banda tiene secuestrado el avión.		
70	La azafata es pelirroja.		
71	El niño está sangrando.		
72	El avión va lleno, no quedan sitios libres en la zona turista.		
73	Mauro se preocupa por el estado del niño.		
74	Antes de aterrizar, Mauro está disfrutando de una bebida con hielo y limón.		
75	Mauro trata de ayudar del niño.		
76	Luisa ordena a los pasajeros que apaguen sus móviles o que se los entreguen enseguida.		
77	Al golpearse el niño y gritar llamando a su madre, los demás pasajeros del avión se asustan.		
78	El comandante informa que regresan al aeropuerto de Madrid-Barajas por motivos de seguridad.		
79	El niño está abrigado con una manta marrón en su asiento de primera clase.		
80	Mauro trata de evitar por todos los medios que los pasajeros negocien con la tripulación y/o hablen con ellos.		

81	Luisa regaña a Mauro por haber agredido a un niño de 5 años.		
82	Cuando Mauro se cambia de asiento, la mujer de al lado tiene puestos los auriculares.		
83	La noticia de que el avión está bajo el control de una banda se extendió por el avión sin que ningún pasajero se pusiera en pie.		
84	El comandante indica que va a aterrizar en el aeropuerto de Miami y pide disculpas por los acontecimientos sucedidos durante el vuelo.		
85	Mauro informa a sus compañeros de revuelta que la tripulación ha pegado a un niño.		
86	El niño no se quita las orejas de Mickey Mouse durante el vuelo.		
87	Luisa se levanta y se dirige a la zona turista, alguien ha tocado el timbre.		
88	Mauro informa al hombre de la perilla que los miembros de la tripulación son los secuestradores.		
89	La mujer del asiento de al lado de Mauro tiene el pelo castaño oscuro.		
90	Suena un teléfono de servicio del avión situado en primera clase.		
91	Antes de aceptar pedir un solo menú, Mauro agarra a Luisa de la mano.		
92	Mauro habla de la rabia, la describe.		
93	La azafata lleva una alianza en la mano izquierda.		
94	Luisa le propina un puñetazo a Mauro.		
95	Uno de los pasajeros de primera clase indica que el vino blanco está muy bueno.		
96	El comandante está jugando al solitario con un ordenador portátil.		
97	Luisa le pide al comandante regresar al aeropuerto de origen, Madrid-Barajas.		
98	Al igual que el resto de pasajeros, Mauro emplea algún utensilio del avión como arma.		
99	Mauro lleva una camisa azul claro y pantalón beige		
100	Luisa informa a Mauro que los pasajeros no pueden acceder a la cabina del comandante.		
101	Por la noche, en las pantallas se proyecta una película.		
102	Mauro se ofrece a solucionar el mal entendido siempre y cuando vuelvan a dar la vuelta al avión en dirección a Miami.		
103	Luisa informa al comandante del incidente entre Mauro y el niño.		
104	Germán lleva a la azafata amordazada con su propio pañuelo.		
105	El comandante llama a la azafata informándola de que hay interferencias en el instrumental de vuelo.		
106	Los dos pilotos tienen el mismo rango, llevan los mismos galones.		
107	El azafato detiene al protagonista evitando que se vaya detrás de la sobrecargo.		

108	Siguiendo a Luisa, Mauro llega hasta la zona reservada para la tripulación.		
109	El grupo de confabuladores disimula al llegar una de las azafatas (vuelven a su asiento).		
110	Luisa comienza a avanzar por el avión sin ver a ningún pasajero.		
111	Uno de los pasajeros es de nacionalidad asiática.		
112	Luisa invita a Mauro a sentarse en su asiento y disfrutar del resto del vuelo (ironía).		
113	El avión está provisto de pantallas.		
114	Mauro intenta parar y hablar con la azafata.		
115	El niño golpea con los pies el asiento de Mauro.		
116	Mauro le entrega a la azafata el móvil con la mano derecha.		
117	Luisa y la azafata llevan perlas.		
118	La madre del niño está viendo algo en la pantalla mientras se bebe una bebida de naranja.		
119	El azafato le pide al protagonista que vuelva a su asiento tal y como le ha indicado Luisa.		
120	La madre del niño lleva vaqueros y una blusa beige.		
121	Luisa lleva unos zapatos de cuña de color azul marino/negro.		
122	El niño lleva unas orejas de plástico de Mickey Mouse.		
123	Mauro elige como menú la pasta.		
124	El copiloto insulta a Mauro por los hechos acontecidos.		
125	Al notificar el comandante que regresan a Madrid, los pasajeros permanecen tranquilos.		
126	Luisa abre la puerta del baño donde está Mauro.		
127	Mauro lee una revista durante la noche.		
128	El comandante y el copiloto llevan uniforme.		
129	La azafata tiene una tirita en el pie izquierdo.		
130	El plan de Mauro consiste en llegar a la cabina y hablar con el comandante.		
131	Le ponen al niño un trozo de papel en la nariz para que pare de sangrar.		
132	Luisa manipula (con el pie) el freno del carrito.		
133	Luisa le comenta al comandante que el niño y su madre van a Disney World.		
134	La corbata del azafato es azul marina.		
135	Mauro miente sobre lo sucedido ("el niño estaba jugando").		
136	Luisa está perpleja, no se cree lo que está pasando.		
137	Los pasajeros de primera clase se unen a la revuelta.		
138	Luisa le explica a Mauro clara y abiertamente por qué dan la vuelta.		
139	Las azafatas llevan una coleta blanca.		
140	Mauro le pide a la azafata que le sirva pasta y pollo.		

141	El menú que ofrece Luisa en el vuelo es de pasta o de pollo.		
142	Luisa le pone una manta al niño.		
143	Muchos pasajeros empiezan a utilizar su teléfono móvil.		
144	El niño lleva un pantalón largo azul marino		
145	Mauro les pide a la azafata y al azafato que se aparten para entrar en la cabina.		
146	La azafata lleva chaleco.		
147	Luisa le dice a uno de los pasajeros de primera clase "lo mejor para los mejores”.		
148	Luisa utiliza gafas de vista para leer.		
149	La madre del niño va al baño.		
150	La chica del asiento contiguo a Mauro pide pasta para comer.		
151	El copiloto tiene perilla.		
152	Germán apoya la idea de Mauro de ir hablar con el comandante.		
153	Mauro suplica y le pide disculpas a Luisa.		
154	Los pasajeros hablan y discuten sobre qué hacer cuando creen que el avión ha sido secuestrado por una banda.		
155	Mauro dice al comandante y copiloto que él ha intentado detener la situación.		

Muchas gracias por su colaboración.
